# Effects of Exposure to Blast Overpressure on Intracranial Pressure and Blood-Brain Barrier Permeability in a Rat Model

**DOI:** 10.1371/journal.pone.0167510

**Published:** 2016-12-01

**Authors:** Usmah Kawoos, Ming Gu, Jason Lankasky, Richard M. McCarron, Mikulas Chavko

**Affiliations:** 1 Department of Neurotrauma, Naval Medical Research Center, Silver Spring, MD, United States of America; 2 Department of Surgery, Uniformed Services University of the Health Sciences and the Walter Reed National Military Medical Center, Bethesda, MD, United States of America; Hungarian Academy of Sciences, HUNGARY

## Abstract

Exposure to blast overpressure (BOP) activates a cascade of pathological processes including changes in intracranial pressure (ICP) and blood-brain barrier (BBB) permeability resulting in traumatic brain injury (TBI). In this study the effect of single and multiple exposures at two intensities of BOP on changes in ICP and BBB permeability in Sprague-Dawley rats was evaluated. Animals were exposed to a single or three repetitive (separated by 0.5 h) BOPs at 72 kPa or 110 kPa. ICP was monitored continuously via telemetry for 6 days after exposure to BOP. The alteration in the permeability of BBB was determined by extravasation of Evans Blue (EB) into brain parenchyma. A significant increase in ICP was observed in all groups except the single 72 kPa BOP group. At the same time a marked increase in BBB permeability was also seen in various parts of the brain. The extent of ICP increase as well as BBB permeability change was dependent on intensity and frequency of blast.

## Introduction

Blast induced traumatic brain injury (bTBI) has received much attention in the past few decades due to an increasing number of military personnel suffering from varying extents of brain injury [1]. TBI is the most prevalent type of injury in personnel involved in combat activity and most of TBI (63%) is caused by explosions[2]. Many war veterans exposed to blast exhibit symptoms of acute and chronic neurological deficits with serious impact on the quality of life and health care. There is insufficient information on the intensity of exposure which may cause mild or moderate TBI [3]. Much effort has been made to understand the biomechanics of TBI caused by blasts in laboratory environment by generating blast pressure waves in shock tubes [4–11]. This has provided a useful model to simulate blast effects and its consequences on physiology, neuropathology, and neurobehavior of animals for investigation in different laboratories [4].

The blood-brain barrier (BBB) is a heterogeneous selective permeability barrier composed of brain endothelial cells and tight junctions, which are pivotal in ensuring the integrity and selectivity of the barrier [12]. The immediate or primary effect of blast induced TBI involves the disruption in cerebral microvasculature and neighboring neuronal cells causing diffuse axonal injury, BBB breakdown, and brain contusions [13]. The delayed or secondary effects (such as inflammation) are initiated at a later point in time as a consequence of the primary damage [13]. The breakdown of BBB has been reported as a characteristic outcome after exposure to blast [12–17]. Skotak el al measured the extent of the BBB breakdown in rats at different levels of blast intensities after exposure to a single blast [18]. They found that the degree of BBB compromise 24 h following exposure to blast closely correlated with the intensity of BOP. In animals exposed to BOP IgG positive cells were seen in brain parenchyma, predominantly in cerebral cortex and hippocampus indicating leakage of markers through the BBB. The primary damage after TBI leads to a cascade of events causing cellular stress, inflammatory response, edema, and changes in intracranial pressure (ICP) [13]. TBI caused by BOP resulted in a transient sharp rise followed by a gradual increase in ICP [9, 18–21]. Interestingly the transient increase in ICP during exposure to a blast wave (138 kPa) generated in shock tube had a higher magnitude than the blast wave itself [19]. Time-course of ICP changes in groups of rats exposed to low-level blasts were reported by Saljo et al [22]. Under low levels of single blast exposure, ICP showed a slow-rising, sustained increase to a maximum level, following which it gradually declined to the normal levels [22]. Saljo et al reported a dependence of increase in ICP (peak and delay in elevation) on the intensity of blast when rats were exposed to a single blast of 10, 30, and 60 kPa [21]. Elevated ICP can be an early response after TBI and in cases where edema and contusions are seen ICP may continue to rise gradually [23]. Clinical experiences suggest that peak ICP elevation after impact TBI may occur 3–5 days after the insult [24]. In a prospective study of 201 TBI patients, intracranial hypertension was documented in 155 patients who had continuous high levels of ICP (> 20 mmHg) lasting at least 5 min [23]. The highest mean ICP was reached within 2 days in one-third of the cases; 3–4 days in another third. By day 5 (after injury) 80% of the cases had reached the maximum mean ICP.

The effects of blast on BBB and ICP also appear to be dose-dependent. It was shown that low intensity blast induces early activation of oxidative and nitrosative reactions, which lead to BBB damage, consequent cerebral inflammation, and increase in ICP [15]. At higher blast intensity levels, BBB disruption seems to occur almost immediately followed by an increase in oxidative stress and the onset of neuroinflammation [8, 25]. Higher intensity blasts result in greater disruption in BBB than low intensity blasts. However the disruption caused by repeated blasts of same intensity may not be additive in nature [26]. In an in vitro BBB model there were no significant differences in the transendothelial electrical resistance (TEER) across a brain endothelial monolayer when comparison was drawn between single and double blasts of the same intensity [26]. Repeated blasting did not significantly reduce TEER, but the second exposure delayed TEER recovery in BBB cultures.

In the present study, TBI caused by blast overpressure (BOP, generated in shock tube) was assessed by monitoring the changes in ICP and BBB permeability in a rat model. The response to single and repetitive blasts at two intensities was characterized.

## Materials and Methods

### Animals and groups

The study protocol was reviewed and approved by the Walter Reed Army Institute of Research/Naval Medical Research Center (NMRC) Institutional Animal Care and Use Committee in compliance with all applicable Federal regulations governing the protection of animals in research. Male Sprague-Dawley rats (weight = 300–350 g and age = 10 weeks at the time of experiment) were obtained from Taconic Farms, NY and were given at least one week to acclimatize in NMRC vivarium. The study was divided into two separate parts to evaluate the effect of blast(s) on ICP and BBB permeability. For each part, the rats were divided into four groups (n = 6 for ICP; n = 6 for BBB) and exposed to either single or repetitive BOP with a peak amplitude (±SD) of 72 ± 3 or 110 ± 3 kPa. The duration of the positive phase (overpressure) and impulse (integral over time) during the positive phase were 5.1 ms and 0.15 kPa.s for 70 kPa; and 7.1 ms and 0.32 kPa.s for 110 kPa blasts. Animals in the repetitive exposure groups were exposed to three consecutive BOPs separated by 0.5 h. A sham group not exposed to blast and treated the same way as the blasted groups (n = 5) was included for BBB permeability study. For ease of presentation, the groups are denoted as: 1x72 kPa, 1x110 kPa, 3x72 kPa, and 3x110 kPa.

### Exposure to BOP

Blast wave was generated in a compressed air-driven shock tube as described before [9, 27]. The static pressure wave characteristics were measured by a piezoelectric sensor (PCB Piezotronics, Buffalo, NY) placed next to the animal’s head. Animals were anesthetized with 5% isoflurane for 3 minutes and secured in a retainer inside the tube in frontal position relative to pressure wave propagation, approximately 1 foot from the open end of the tube.

### ICP monitoring

ICP was measured by Millar telemetry system [28] (Millar Instruments. Inc., Houston, TX) in freely moving animals, except for 3 min at the time of BOP exposure. The system consists of an ICP telemeter for pressure measurement and wireless transmission; SmartPad as a power supply and a wireless link to the telemeter; and PowerLab and LabChart (ADInstruments, Colorado Springs, CO) for data acquisition from SmartPad, recording and display. ICP data was collected at a sampling rate of 1k/s and a triangular window (n = 33) was used for smoothing. The telemeter is completely implantable and consists of a sensor-tipped catheter emanating from a body holding the electronics. The telemeter is designed in such a way that the sensor is placed in a target location and the body is implanted in the abdominal sac of the animal. The positioning of the telemeter body ensures adequate battery charging and seamless data transmission when the animal is placed on top of the SmartPad. For continuous monitoring of ICP, animals underwent a sterile surgical procedure for the implantation of ICP telemeter.

#### ICP telemeter implantation

Animals were anesthetized with a mixture of Ketamine/Xylazine (i.p., 70/4 mg/kg). A 2 cm incision was made in the lower right quadrant of the abdomen and the telemeter body was inserted behind the viscera and sutured to the internal abdominal wall. The sensor-tipped catheter was tunneled through the subcutaneous space over animal’s back and neck to later emerge from the incision made on its head. The abdominal incision was sutured and the animal was turned to the ‘sphinx’ position. The animal’s head was immobilized in a stereotaxic frame and a 1 cm incision was made on the dorsal midline of the scalp. The skin was removed to expose the bregma. A 1 mm hole was drilled in the bone using a tapered dental burr at 0.9 mm lateral from midline and 1.5 mm posterior to bregma. A 25-gauge needle was used to puncture dura for insertion of the pressure sensor-tipped catheter. The sensor was inserted to reach a depth of 3.5 mm below the surface of the skull in order to be positioned in the right lateral ventricle. The catheter was glued to the surface of the skull by Vetbond ™ (cyanoacrylate, Hanna Pharmaceuticals Supply Co. Inc., Wilmington, DE) and the scalp incision was sutured. After surgery animals received Ketorolac (5 mg/kg, subcutaneously), and they were allowed to recover from anesthesia. The proper placement of sensor and the response of telemeter were confirmed by observing an increase in ICP after compressing internal jugular vein and/or suspending the animal by its tail with the head in ‘dependent’ position. A period of 24 h was allowed for the stabilization of the telemeter before animals were exposed to blast. Animals were randomly assigned to groups for exposure to blast and ICP was continuously monitored for 6 days after the blast. At the end of study animals were euthanized with Euthasol (Virbac AH, Inc., Fort Worth, TX).

#### Data analysis

For simplification of data analysis, representative data spanning over a time window of 3 h (Day -1, pre-blast; Day 1—Day 5, post-blast) or 4 h (Day 0, day of blast) were selected, with each window starting at the same time of day. The data are expressed as means ± SE and two-way ANOVA followed by Bonferroni post-hoc tests (with p<0.05 considered to be significant) was used for (calculating) statistical significance. Full 7 day ICP telemetry data are provided as supplementary information (S1–S4 Files).

### BBB permeability evaluation

Effect of exposure to BOP on BBB permeability was determined by the leaking of permeability marker Evans blue (EB) into brain parenchyma [11]. EB (4%, 1 ml/kg) was administered through the tail vein of rats 24 h after exposure to blast. The animals were allowed to recover for 2 hours, anesthetized with Euthasol and perfused transcardially with 200 ml of saline. Brains were removed, post-fixed in 0.1 M phosphate buffer containing 4% paraformaldehyde, and transferred to 20% sucrose overnight. The sham group was treated similarly as blast groups with the exception of BOP exposure.

The harvested brains were sectioned into 1.5 mm-thick coronal slices, rostral to caudal, using a brain matrix [29] taken from 4 mm anterior to bregma for frontal cortex (FCX), 3.5 mm posterior to bregma for hippocampus (HIP), 5 mm posterior to bregma for thalamus (THL), and 8 mm posterior to bregma for occipital cortex (OCX) to quantify the EB presence. EB labeling in brain areas was examined with a Nikon fluorescence microscope under red fluorescence [30]. The images of brain sections were analyzed with Pro Image J Plus software. The regions of interest (ROI) where the fluorescent density of EB appeared to be significant in FCX, HIP, THL, and OCX were selected from the image of a brain section. For each animal the optical density (OD) for a given region was an average taken over the ROIs from 2 or 3 brain sections while attention was paid to maintain consistency in the sizing and positioning of the ROIs. The OD for BOP groups was normalized with respect to a sham group by dividing the average OD from an animal from BOP group by the average OD of the sham group. Five animals of sham group and six animals of each BOP groups were used for statistical analysis, group data was compared using one-way ANOVA and Fisher’s test for multiple comparison with p<0.05 considered to be significant.

## Results

### ICP study

**Fig 1:**
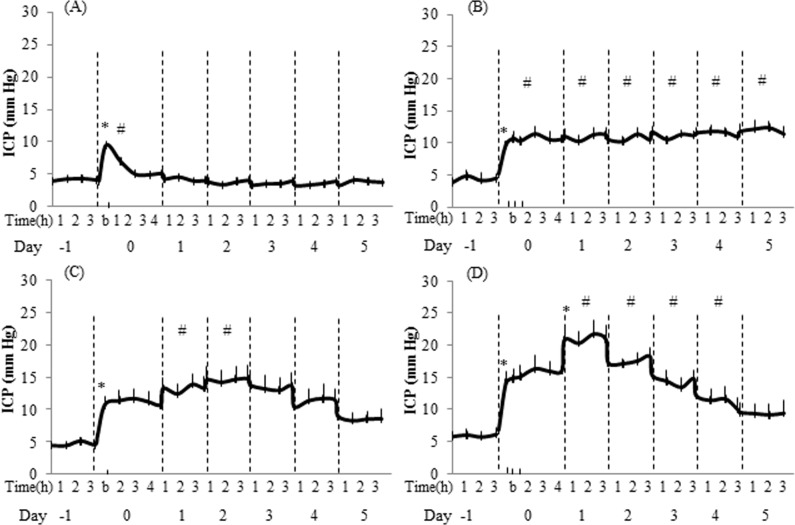
Time course of changes in ICP in response to exposure to BOP. For each day (marked with vertical dashed lines) a three or four hour segment of data is presented. A) 1x72 kPa; B)3x72 kPa; C) 1x110 kPa; and D) 3x110 kPa. Data are expressed as means ± SE (n = 6 in each group). b—time of blast; * p<0.01—significance of differences in ICP between two adjacent time points, # p<0.01—significance of differences in ICP compared to pre-blast baseline.

The baseline values of ICP remained stable and in the physiological range prior to the exposure to BOP. Fig 1 shows the changes in ICP recorded during a five day interval in four groups exposed to BOP. There are two patterns in the ICP change as a result of exposure to BOP. First, the magnitude of ICP increase is dependent on the intensity of BOP, with higher intensity causing greater increase in ICP. Second, the increase in ICP depends on the number of blast exposures, single vs repetitive, showing significant differences in the kinetics of the change in ICP. At a lower intensity of blast (72 kPa), a single blast exposure causes a short-term elevation in the ICP which returns to near baseline values on the day of blast (Fig 1A). At the same time repetitive exposure to 72 kPa resulted in a steady increase in ICP over the period of study (Fig 1B). In single or repeated exposure to 110 kPa, ICP increased on the day of blast and gradually reached a maximum, followed by a decline towards a baseline. The ICP pattern for the two groups exposed to 110 kPa BOP differed in two respects- the ICP peak magnitude and the time duration to reach the peak. Compared with a single exposure, a higher ICP peak magnitude and a shorter delay to reach the peak were observed in the repetitive blast group (Day 1 vs. Day 2, Fig 1C and 1D).

### BBB permeability evaluation

**Fig 2:**
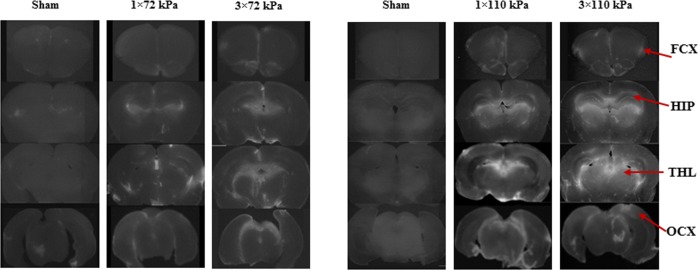
Representative images of EB fluorescence in brains of sham-controls and rats exposed to single or repetitive BOP at two different intensities (72 kPa vs 110 kPa). Sections of brain were analyzed for EB fluorescence in frontal cortex (FCX), hippocampus (HIP), thalamus (THL), and occipital cortex (OCX). The EB fluorescence was increased after exposure to BOP suggesting higher vascular leakage into brain parenchyma.

**Fig 3:**
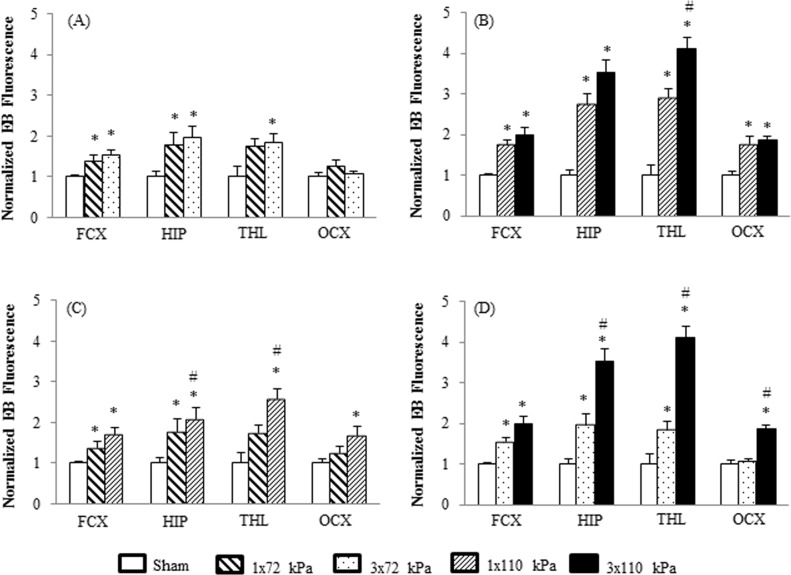
Quantification of EB fluorescence intensity in sham and blast exposed rats. Results are shown as relative EB fluorescence units (controls = 1) and are expressed as means ± SE (n = 6). A) Comparison between groups 1x72 kPa and 3x72 kPa; B) 1x110 kPa and 3x110 kPa; C) 1x72 kPa and 1x110 kPa; D) 3x72 kPa and 3x110 kPa. *p<0.05—difference between sham and BOP exposed groups. #p<0.05—difference between BOP exposed groups. FCX: frontal cortex; HIP: hippocampus; THL: thalamus; OCX: occipital cortex.

The morphology of vascular leakage in the brain of rats exposed to BOP is shown in Fig 2. Compared with sham group certain areas of the brain exposed to BOP exhibited higher EB fluorescence, suggesting higher vascular leakage of the marker into brain parenchyma. The EB fluorescence density was analyzed in the brain areas (shown in Fig 2) to quantitatively determine presence of the marker in brain after single or repeated exposure to BOP at two different intensities. Compared with sham, the EB fluorescence was significantly increased after exposure to both BOP intensities across the brain. At low intensity (72 kPa) there was an increase of EB fluorescence in all brain regions except for thalamus and occipital cortex, and fluorescence did not significantly differ between single or multiple exposure (Fig 3A). At the higher blast level fluorescence was increased in all brain regions including occipital cortex and the EB fluorescence was significantly higher in thalamus of rats exposed to repetitive versus single BOP (Fig 3B). When comparing the effect of two intensities of BOP on vascular leakage it was found that 110 kPa blasts (either single or repetitive) induced higher increase of EB fluorescence in hippocampus, thalamus, and occipital cortex compared with 72 kPa BOP (Fig 3C and 3D).

## Discussion and Conclusion

The main finding of this study was identifying a qualitative relationship between changes in ICP and BBB integrity with the intensity and frequency of BOP. It was shown that the magnitude of change in ICP was directly proportional to the intensity and frequency of BOP. At low intensity single blast exposure (72 kPa), ICP remained elevated for a short time on the day of blast and shortly thereafter returned to near baseline level. However, three repetitive exposures to 72 kPa BOP resulted in a sustained increase in ICP without return to baseline during the period of 6 days following exposure. In the higher intensity blast groups (110 kPa), the magnitude of ICP increase was higher compared with the low intensity groups. Moreover, the time course and magnitude of ICP change was different in single and repetitive exposure. In this scenario, single blast exposure resulted in a longer time to peak ICP and a lower ICP peak magnitude in comparison to repetitive blasts. It could be suggested that the ‘high intensity repetitive’ paradigm results in an early activation of the secondary injury reactions following primary injury in comparison to the lower intensity BOP with a relatively slower increase in ICP.

In general, detection of the EB fluorescence inside the brain parenchyma as a marker of BBB leakage corresponds with changes in the ICP after exposure to BOP. It was reported that the earliest pathological findings after 24 h of exposure to blast are the presence of blood in ventricles and choroid plexus [31, 32]. This occurs even after exposures to mild blast intensities (74.5 kPa) which can be attributed to the direct shearing effects of blast on the choroid plexus leading to vascular rupture and leakage of blood into the ventricles. Consistent with previous studies, our results suggested EB fluorescence surrounding ventricle and corpus callosum may be associated with blast-induced direct vascular damage. Confocal images have shown strong widespread EB fluorescence in cortex after blast [14] and the optical density of EB in frontal cortex of blast group was over 60% higher than the sham group [11]. It was reported that focal lesions were present in many brain regions including the cortex, which were likely caused by direct shear effects induced by blast [31, 32]. Both, changes in ICP and EB leakage were related to the BOP intensity with a more pronounced effect observed in multiple exposures to the higher levels of BOP. While EB is a commonly used marker for the assessment of BBB integrity, its use is limited by some disadvantages such as its non-specific binding to albumin and potential presence of free dye in the brain tissue [33]. Other markers for the determination of BBB permeability, such as radiolabeled compounds, sodium fluorescein (NaFl), and dextrans are less subject to these limitations. In particular, biotin and fluorophor labeled dextrans are valuable markers of BBB integrity as they appear to be non-toxic in small quantities, have not been reported to bind with proteins or tissue, and can be visualized under light or electron microscopy [33]. Nonetheless, a study using systemic administration of NaFl, EB, and dextrans to estimate the pore size of BBB opening and the time required for recovery in an *in vivo* bTBI model, [26] revealed no difference in the time course of extravasation of all three markers in the brain after exposure to blast. However, it appears that NaFl and dextran extravasation was higher in some brain regions compared with EB, indicating better permeability and detection sensitivity. Despite of some limitations the similar time course of extravasation of the three markers supports the use of EB as a rapid method for determination of a BBB leak.

Previous studies reported that TBI induced by closed-head [34] and fluid percussion injury[35] in rats resulted in immediate (30 min) and late (6–10 h) rises in ICP [34, 36]. The late increase was followed by a slow decline toward control levels, which was reached in about a week [35]. The immediate increase is supposed to be vascularly mediated and is followed by a temporary decrease of ICP, which can be attributed to the return of vascular control [34]. The late ICP increase was accompanied by decrease in cerebral blood flow and metabolic rate of oxygen, presumably secondary to brain edema [37] and/or to other secondary mechanisms of injury [34]. Similar to our results, a previous study demonstrated a dose dependent effect on ICP after exposure to 10, 30, and 60 kPa BOP [21]. At the same time, the initial elevation was delayed after exposure to lower levels of BOP. In all exposures, ICP returned to control levels after 7 days. We demonstrated that at repetitive exposure to the higher blast intensity (110 kPa), the baseline ICP remained elevated and did not return to the pre-blast baseline. The increase in ICP has also some implication for behavioral and cognitive function as the latency to reach the platform assessed with the Morris water maze was increased by over 100% after blast [21]. Further experiments are needed to establish the direct relationship between ICP increase and intensity of exposure to BOP for its possible use as a marker of BOP-induced brain damage.

The brain is enclosed in an inflexible bony skull and for ICP to remain stable the total intracranial volume (brain, blood, and CSF) must remain constant. During TBI fluids can move from one space to another with no net significant change in the total contents of the cranial vault. This transfer of fluids does not contribute significantly to brain edema formation and intracranial hypertension. However an increase in BBB permeability can be a passive driving force for the build-up of cytotoxic edema [38] and may also lead to vasogenic edema [39], both of which, in turn, could contribute to post-traumatic intracranial hypertension. An increase in ICP may impede cerebral blood flow leading to cerebral ischemia and secondary brain injury. Elevated ICP becomes more predominant when cerebrospinal fluid regulation is impaired and the permeability of the BBB is increased. Blast pressure waves can have a direct effect on the brain by mechanical forces, leading to an increase in ICP with secondary effects on brain functions such as impairment of cognition, motor activity, or peripheral auditory and vestibular system function. From a therapeutic point-of-view, it is critical to understand this inter-relationship and determine a correlation between the various stages of these processes. Brain injury is an outcome of a complex process involving interdependence of causative processes. The management of brain injury requires novel approaches for controlling ICP and for designing trials and treatment of TBI. The degree and duration of elevation in ICP is associated with the outcome after TBI. The maintenance of stable ICP and cerebral perfusion pressure are fundamental therapeutic goals after TBI [40].

Exposure to BOP is known to initiate activation of cell damaging biochemical mechanisms due to stress waves and acute mechanical forces. Another potential mechanism includes indirect mechanisms of damage by pressure wave transmission through blood and CSF [41]. The potential target for damage by stress waves, mechanical forces or blood pressure changes is the cerebral vasculature [15]. Initial damaging mechanisms may involve activation of free radical generating enzymes leading to oxidative and nitrosative damage; and damage to tight junction proteins of the endothelial cells. A literature review shows that there is decrease of tight junction proteins such as occludin, claudin-5, zonula occluden 1 (ZO-1) after TBI including blast-brain injury [12, 15, 42–44]. Using immunofluorescence staining and Western blotting, expression of those tight junction proteins was found to be significantly decreased by single or repeated BOP of 123 kPa. It was suggested that the oxidative stress induced by BOP evolves into disruption of the BBB influx of proteins such as albumin, fibrinogen, and thrombin, which together may contribute to microglial activation, oxidative stress, and the release of proinflammatory mediators in the brain [15]. Kabu et al reported an escalation in the generation of reactive oxygen species (ROS) after exposure to shock waves [11]. The increase in ROS levels and cell apoptosis was directly proportional to the blast intensity and the time after blast exposure, suggesting a sustained neuronal injury response. Other mechanisms of damage may include oxidative-stress induced activation of matrix metalloproteinases and fluid channel receptor aquaporin-4, facilitating vascular fluid transfer, edema, and leakage of the BBB followed by neuroinflammation. In our preliminary experiments a single exposure to 72 kPa blast resulted in decreased immunoreactivity of the tight junction protein occludin in the cerebral vessels of prefontal cortex and increased nitrotyrosine immunoreactivity in the cortex when compared with non-blast control rats (data not presented here). It was reported by others that at low intensity BOP exposure(s), oxidative stress precedes alterations in BBB integrity and neuroinflammation [15, 43]. On the contrary, the mechanical rupture of cerebral microvasculature is an immediate response to high intensity BOP exposure(s), followed by oxidative stress [8, 25, 43].

Numerous studies have shown that exposure to BOP alters the integrity of BBB [11, 15, 43, 45]. This can result from increased vascular leakage due to disruption in BBB integrity as a consequence of mechanical force interaction with brain [11]. This is supported by a positive correlation between the degree of vascular leakage and the intensity of shock waves [11]. Recently, it was suggested that the vulnerability of the vasculature to impairment might be a selective type of damage caused by low level BOP as microvascular pathology was reported in otherwise normal brain parenchyma [46]. A more recent study showed that repetitive BOP exposure (three daily 105 kPa BOPs) induced a distinctive pattern of regionally and temporally restricted disruption in the cerebral vasculature [47]. These disturbances lead to an evolving insult to the central nervous system which functions as a precursor to long-lasting extensive neuroinflammatory responses. Sustained activation of immune response following vascular injury may result in chronic neurocognitive changes. A review [43] of the mechanism of BBB dysfunction following BOP suggests that lower intensity blast exposures induce oxidative and nitrosative stress followed by a cascade of processes [15]. Free radical scavengers and antioxidants were found to be neuroprotective in other TBI models [48]. So far, effective pharmacological interventions to prevent oxidative damages after blast induced TBI that may attenuate breakdown in BBB, edema formation, increase in ICP, and progression of neurocognitive and behavioral impairment in humans remains to be found.

In conclusion, the magnitude and time course of relationship between changes in ICP and BBB permeability and intensity and frequency of exposure to BOP were studied in a rat model. Results showed a direct correlation between intensity of BOP exposure and both, ICP increase and extravasation of EB. In addition, results showed that multiple exposures significantly aggravated the effect of single exposure to BOP. The correlation between BBB opening and ICP increase could provide still missing information about threshold values for brain damage depending on intensity and frequency of BOP with potential diagnostic value.

## Supporting Information

S1 File Full 7 day ICP telemetry data for 1x72 kPa group. (PDF)

S2 File Full 7 day ICP telemetry data for 3x72 kPa group. (PDF)

S3 File Full 7 day ICP telemetry data for 1x110 kPa group. (PDF)

S4 File Full 7 day ICP telemetry data for 3x110 kPa group. (PDF)
