# Supplementary material for: Effects of Exposure to Blast Overpressure on Intracranial Pressure and Blood-Brain Barrier Permeability in a Rat Model
Source: PLoS One. 2016 Dec 1;11(12):e0167510. doi: 10.1371/journal.pone.0167510 (PMC5132256; doi:10.1371/journal.pone.0167510)
Supplement: S1 File — (PDF) [file pone.0167510.s001.pdf]

Full 7 day telemetry ICP (intracranial pressure) data for 1x72 kPa group. The highlighted data is presented in Fig 1A.

			ICP (mmHg)							
		Time	Animal number							
Day	Event	hh:mm	1	2	3	4	5	6	Average	SE
-1	Baseline	9:00	4.56	4.34	4.76	4..28	4.53	4.76	4.59	0.07
		**10:00**	**4.11**	**4.26**	**4.28**	**3.19**	**3.55**	**4.49**	**3.98**	**0.20**
		**11:00**	**3.47**	**4.85**	**4.66**	**3.96**	**4.05**	**4.84**	**4.31**	**0.23**
		**12:00**	**2.81**	**4.27**	**5.2**	**4.69**	**3.36**	**5.75**	**4.35**	**0.45**
		**13:00**	**4.03**	**3.63**	**4.52**	**4.25**	**3.11**	**5.26**	**4.13**	**0.30**
		14:00	4.06	4.87	3.92	4.38	3.53	5.50	4.38	0.29
		15:00	4.85	4.72	4.62	4.00	3.56	4.69	4.41	0.21
		16:00	4.32	4.49	4.80	4.62	3.93	5.18	4.56	0.17
		17:00	4.70	4.16	4.05	4.22	3.53	5.53	4.37	0.28
		18:00	4.28	4.78	5.02	4.03	3.58	5.12	4.47	0.25
		19:00	4.38	4.33	4.71	4.00	3.90	4.97	4.38	0.17
		20:00	4.38	3.76	4.09	4.57	3.72	4.79	4.22	0.18
		21:00	4.92	3.97	4.42	3.69	3.67	5.08	4.29	0.25
		22:00	4.39	4.30	4.77	4.28	3.98	4.60	4.39	0.11
		23:00	4.10	4.70	4.68	4.11	3.84	5.25	4.45	0.21
0		0:00	4.28	4.69	3.57	4.35	3.87	5.17	4.32	0.23
		1:00	4.17	3.74	4.36	4.17	3.36	5.00	4.13	0.23
		2:00	4.25	4.77	4.16	4.63	3.97	4.68	4.41	0.13
		3:00	4.53	3.86	4.04	4.34	3.64	4.38	4.13	0.14
		4:00	4.57	4.10	5.02	4.38	3.91	5.43	4.57	0.23
		5:00	4.27	3.71	3.83	3.87	3.79	5.51	4.16	0.28
		6:00	4.40	4.16	4.90	3.78	4.01	4.54	4.30	0.16
		7:00	4.52	4.70	4.91	4.40	3.88	5.21	4.60	0.19
		8:00	4.81	4.13	4.58	4.36	3.70	5.15	4.46	0.21
		9:00	3.96	3.95	4.05	4.32	3.56	4.05	3.98	0.10
	Blast 1	**10:00**	**10.01**	**7.77**	**9.43**	**9.1**	**9.65**	**11.16**	**9.52**	**0.45**
		**11:00**	**7.23**	**6.01**	**7.2**	**7.93**	**7.2**	**6.19**	**6.96**	**0.30**
		**12:00**	**6.26**	**3.07**	**5.26**	**5.37**	**3.62**	**6.51**	**5.02**	**0.57**
		**13:00**	**6.45**	**4.93**	**3.52**	**3.91**	**5.87**	**5.02**	**4.95**	**0.46**
		**14:00**	**6.81**	**5.34**	**4.48**	**4.91**	**4.2**	**4.95**	**5.12**	**0.38**
		15:00	7.69	7.05	6.63	6.04	4.62	5.69	6.29	0.44
		16:00	6.85	6.62	7.40	5.30	4.40	5.36	5.99	0.47
		17:00	4.91	6.11	5.45	5.39	4.65	5.69	5.37	0.22
		18:00	4.72	6.50	4.55	6.09	4.82	5.29	5.33	0.33
		19:00	4.53	6.36	5.02	5.07	4.63	5.57	5.19	0.28
		20:00	4.77	4.47	5.41	4.98	4.81	5.15	4.93	0.13
		21:00	5.78	4.65	7.32	6.27	5.49	5.37	5.81	0.37
		22:00	4.38	5.43	5.01	4.59	4.39	4.66	4.74	0.17
		23:00	4.65	6.28	5.59	5.82	5.49	5.21	5.51	0.23

					ICP (mmHg)					
		Time			Animal number					
Day	Event	hh:mm	1	2	3	4	5	6	Average	SE
1		0:00	4.48	5.64	4.58	4.39	4.75	4.56	4.73	0.19
		1:00	4.61	6.50	4.86	4.73	4.68	4.49	4.98	0.31
		2:00	5.74	5.62	5.08	4.71	4.66	4.68	5.08	0.20
		3:00	4.78	6.05	4.78	4.70	4.34	4.38	4.84	0.26
		4:00	6.21	5.90	4.80	4.62	4.36	4.62	5.08	0.32
		5:00	4.97	4.53	5.29	4.57	4.40	4.43	4.70	0.14
		6:00	5.48	4.87	5.21	4.48	4.73	4.73	4.92	0.15
		7:00	6.49	4.83	4.83	4.70	4.65	4.48	5.00	0.30
		8:00	6.42	5.40	4.69	4.54	4.62	4.63	5.05	0.30
		9:00	5.08	6.14	4.57	4.45	4.67	4.44	4.89	0.27
		**10:00**	**4.14**	**5.18**	**3.96**	**4.82**	**3.39**	**4.2**	**4.28**	**0.26**
		**11:00**	**6.37**	**5.82**	**2.91**	**3.57**	**4.62**	**4.22**	**4.59**	**0.54**
		**12:00**	**3.75**	**3.8**	**4.98**	**3.95**	**3.44**	**5.55**	**4.25**	**0.34**
		**13:00**	**3.38**	**3.99**	**4.09**	**4.35**	**4.15**	**4.19**	**4.03**	**0.14**
		14:00	4.58	4.41	4.57	3.92	5.04	4.62	4.52	0.15
		15:00	4.33	4.63	4.35	4.10	5.10	3.14	4.28	0.27
		16:00	4.69	3.78	3.83	4.72	4.77	5.12	4.48	0.22
		17:00	4.53	4.73	4.08	2.84	4.52	3.92	4.10	0.28
		18:00	4.72	3.52	3.98	4.64	5.30	5.27	4.57	0.29
		19:00	4.41	4.14	3.16	4.54	4.37	3.80	4.07	0.21
		20:00	4.64	4.01	4.71	3.34	5.40	4.28	4.40	0.28
		21:00	4.60	3.46	4.74	3.20	5.60	3.13	4.12	0.41
		22:00	4.68	4.58	3.74	2.85	4.72	5.51	4.35	0.38
		23:00	4.58	4.57	4.26	4.33	4.72	3.53	4.33	0.18
2		0:00	4.74	4.69	3.67	3.79	4.34	4.50	4.29	0.19
		1:00	4.43	4.21	4.20	4.02	4.83	3.25	4.16	0.21
		2:00	4.60	4.04	3.94	2.99	4.42	4.85	4.14	0.27
		3:00	4.53	4.21	3.43	4.49	4.24	5.28	4.37	0.24
		4:00	4.67	3.75	3.43	4.73	5.35	4.73	4.44	0.29
		5:00	4.45	3.50	4.74	4.48	4.10	4.12	4.23	0.18
		6:00	4.66	3.84	3.53	3.29	4.55	4.19	4.01	0.22
		7:00	4.44	3.65	3.26	3.68	5.35	4.28	4.11	0.30
		8:00	4.73	2.98	3.80	3.79	4.79	5.31	4.23	0.35
		9:00	4.42	2.71	4.44	3.12	5.49	4.88	4.18	0.43
		**10:00**	**4.33**	**2.76**	**3.36**	**3.45**	**4.81**	**4.33**	**3.84**	**0.31**
		**11:00**	**3.27**	**2.95**	**3.39**	**2.55**	**5.36**	**2.85**	**3.40**	**0.41**
		**12:00**	**4.94**	**2.67**	**4.09**	**2.52**	**4.03**	**4.73**	**3.83**	**0.42**
		**13:00**	**3.28**	**3.78**	**4.76**	**2.73**	**5.9**	**3.83**	**4.05**	**0.46**
		14:00	5.16	3.63	4.50	3.70	5.33	4.02	4.39	0.30
		15:00	4.76	3.50	4.03	3.27	5.38	4.72	4.28	0.33
		16:00	3.72	3.68	4.28	3.73	5.01	4.59	4.17	0.23
		17:00	4.80	3.22	4.72	3.17	5.19	4.04	4.19	0.35
		18:00	3.35	3.51	4.04	3.69	5.65	4.30	4.09	0.34
		19:00	3.89	3.57	4.38	3.20	4.57	4.52	4.02	0.23

			ICP (mmHg)							
		Time	Animal number							
Day	Event	hh:mm	1	2	3	4	5	6	Average	SE
		20:00	3.99	3.54	4.26	3.10	4.96	4.53	4.06	0.28
		21:00	4.88	3.27	4.65	2.99	5.73	4.37	4.31	0.42
		22:00	5.14	3.37	4.74	3.66	4.82	3.51	4.21	0.32
		23:00	5.01	3.64	4.22	3.70	5.28	4.05	4.32	0.28
3		0:00	4.79	3.48	4.28	3.70	4.71	3.86	4.14	0.22
		1:00	4.08	3.31	4.68	3.25	4.58	3.92	3.97	0.25
		2:00	5.23	3.65	4.36	3.63	5.36	3.87	4.35	0.32
		3:00	5.07	3.54	4.18	3.89	4.88	3.90	4.24	0.25
		4:00	4.87	3.59	4.63	3.02	5.52	4.01	4.27	0.37
		5:00	3.84	2.97	4.26	3.44	5.37	4.12	4.00	0.33
		6:00	4.76	3.07	3.97	3.22	5.12	4.66	4.13	0.35
		7:00	3.01	3.71	3.99	3.62	5.38	4.32	4.01	0.33
		8:00	3.51	3.38	4.54	3.70	5.57	4.53	4.20	0.34
		9:00	3.24	3.00	4.66	3.05	4.72	3.94	3.77	0.32
		**10:00**	**2.76**	**2.88**	**3.93**	**3.37**	**3.53**	**3.66**	**3.36**	**0.19**
		**11:00**	**3**	**2.82**	**4.09**	**3.41**	**4.06**	**3.85**	**3.54**	**0.22**
		**12:00**	**3.96**	**2.66**	**4.31**	**3.26**	**4.26**	**2.98**	**3.57**	**0.29**
		**13:00**	**4.06**	**4.17**	**4.38**	**3.59**	**4.57**	**3.01**	**3.96**	**0.23**
		14:00	4.03	3.40	4.20	4.01	3.42	3.02	3.68	0.19
		15:00	3.19	3.86	4.45	4.00	4.41	3.16	3.84	0.23
		16:00	3.73	3.29	4.47	3.66	3.50	3.94	3.77	0.17
		17:00	3.89	3.21	4.51	4.07	4.00	3.70	3.90	0.18
		18:00	3.75	3.57	4.57	3.81	3.24	4.41	3.89	0.21
		19:00	3.71	3.82	4.47	3.93	3.70	3.21	3.81	0.17
		20:00	4.03	3.74	4.16	4.63	4.41	3.24	4.04	0.20
		21:00	3.14	3.40	4.14	3.87	3.27	4.17	3.66	0.18
		22:00	3.28	3.47	4.17	3.42	2.96	3.12	3.40	0.17
		23:00	3.91	3.72	4.08	3.96	3.53	4.01	3.87	0.08
4		0:00	3.11	3.09	4.16	3.21	2.94	4.34	3.47	0.25
		1:00	4.03	3.29	4.24	4.05	4.35	4.48	4.07	0.17
		2:00	3.54	3.71	4.06	4.64	4.43	3.10	3.91	0.23
		3:00	3.74	3.86	4.08	3.36	3.00	4.38	3.74	0.20
		4:00	4.03	3.53	4.28	3.12	3.85	4.17	3.83	0.18
		5:00	3.53	3.53	4.43	3.67	3.93	3.46	3.76	0.15
		6:00	3.64	3.94	4.31	4.73	3.05	3.04	3.79	0.28
		7:00	3.28	3.68	4.14	4.33	4.07	3.32	3.80	0.18
		8:00	3.28	3.57	4.33	4.37	3.54	4.00	3.85	0.18
		9:00	3.84	3.99	4.09	3.68	3.42	3.44	3.75	0.11
		**10:00**	**2.8**	**2.22**	**3.96**	**2.91**	**4.41**	**3.16**	**3.24**	**0.33**
		**11:00**	**3.4**	**3.15**	**4.06**	**3.4**	**2.94**	**3.28**	**3.37**	**0.15**
		**12:00**	**3.13**	**2.88**	**4.18**	**3.94**	**4.42**	**3.17**	**3.62**	**0.26**
		**13:00**	**4.25**	**3.7**	**3.35**	**4.6**	**4.56**	**2.95**	**3.90**	**0.28**
		14:00	4.13	3.86	4.16	3.76	4.76	3.49	4.03	0.18
		15:00	3.98	3.46	4.56	3.89	4.53	4.16	4.10	0.17

		Time			ICP (mmHg) Animal number					
Day	Event	hh:mm	1	2	3	4	5	6	Average	SE
		16:00	3.34	3.08	4.06	3.47	3.998	4.29	3.71	0.19
		17:00	3.06	3.89	3.94	4.23	4.307	4.53	3.99	0.21
		18:00	3.59	3.41	4.46	3.84	4.7	4.58	4.10	0.23
		19:00	3.95	3.00	4.45	3.64	4.008	4.58	3.94	0.23
		20:00	3.57	4.06	4.45	3.60	4.655	4.47	4.13	0.19
		21:00	3.39	3.70	4.58	3.64	4.354	4.10	3.96	0.19
		22:00	3.98	2.95	4.35	3.67	4.40	4.14	3.91	0.22
		23:00	3.23	3.73	4.56	3.34	4.70	4.52	4.01	0.27
5		0:00	3.74	3.40	4.57	3.51	4.78	4.28	4.05	0.24
		1:00	3.18	3.20	4.39	3.70	4.12	4.17	3.79	0.21
		2:00	3.32	3.80	4.56	4.74	4.00	4.40	4.14	0.22
		3:00	3.21	2.96	4.31	3.81	4.30	4.47	3.84	0.26
		4:00	3.13	3.01	4.19	4.38	4.54	4.45	3.95	0.28
		5:00	3.68	3.08	4.06	3.51	4.16	4.21	3.78	0.18
		6:00	2.91	3.63	4.54	3.76	3.94	4.13	3.82	0.22
		7:00	3.79	3.77	4.46	4.61	4.20	4.16	4.16	0.14
		8:00	3.51	3.03	4.06	3.87	4.58	4.68	3.96	0.26
		9:00	3.83	3.73	3.98	4.32	4.50	4.01	4.06	0.12
		**10:00**	**2.38**	**2.39**	**3.34**	**3.63**	**3.67**	**4.14**	**3.26**	**0.30**
		**11:00**	**4.05**	**2.99**	**4.78**	**4.77**	**4.17**	**3.93**	**4.12**	**0.27**
		**12:00**	**3.64**	**3.1**	**5.79**	**4.15**	**3.6**	**3.21**	**3.92**	**0.40**
		**13:00**	**2.18**	**2.96**	**4.16**	**4.55**	**3.93**	**4.83**	**3.77**	**0.41**
		14:00	3.46	3.49	5.42	4.76	4.59	3.98	4.28	0.32
		15:00	3.85	4.16	4.86	4.39	4.35	4.23	4.31	0.14
