# Supplementary material for: Effects of Exposure to Blast Overpressure on Intracranial Pressure and Blood-Brain Barrier Permeability in a Rat Model
Source: PLoS One. 2016 Dec 1;11(12):e0167510. doi: 10.1371/journal.pone.0167510 (PMC5132256; doi:10.1371/journal.pone.0167510)
Supplement: S2 File — (PDF) [file pone.0167510.s002.pdf]

Full 7 day telemetry ICP (intracranial pressure) data for 3x72 kPa group. The highlighted data is presented in Fig 1B.

					ICP (mmHg)					
		Time			Animal number					
Day	Event	hh:mm	1	2	3	4	5	6	Average	SE
-1	Baseline	9:00	3.46	3.42	5.46	4.56	2.86	5.46	4.20	0.46
		**10:00**	**2.32**	**2.87**	**6.02**	**4.3**	**1.87**	**5.9**	**3.88**	**0.74**
		**11:00**	**3.84**	**2.89**	**8.35**	**4.9**	**3.08**	**6.43**	**4.92**	**0.87**
		**12:00**	**1.65**	**1.95**	**6.85**	**4.92**	**1.26**	**8.37**	**4.17**	**1.23**
		**13:00**	**3.08**	**4.7**	**4.73**	**6.09**	**2.48**	**6.19**	**4.55**	**0.62**
		14:00	3.67	4.21	5.25	5.23	3.33	6.84	4.76	0.53
		15:00	3.75	4.16	6.71	5.60	3.08	6.83	5.02	0.65
		16:00	3.75	4.77	6.75	4.89	3.79	5.39	4.89	0.46
		17:00	3.61	4.36	6.19	4.23	3.27	7.08	4.79	0.62
		18:00	3.58	3.50	5.82	5.24	3.84	6.28	4.71	0.50
		19:00	3.81	4.24	6.73	4.09	3.17	5.18	4.54	0.51
		20:00	3.57	4.23	6.56	5.36	3.16	7.24	5.02	0.67
		21:00	3.81	4.45	6.46	4.55	3.74	6.50	4.92	0.51
		22:00	3.64	3.13	5.41	4.58	3.57	6.25	4.43	0.49
		23:00	3.77	3.57	6.18	5.55	3.83	5.27	4.70	0.45
0		0:00	3.57	4.49	6.39	4.32	3.26	5.75	4.63	0.50
		1:00	3.65	4.76	6.56	4.32	3.63	7.69	5.10	0.68
		2:00	3.80	4.57	5.26	4.56	3.32	7.57	4.85	0.61
		3:00	3.83	3.34	6.37	5.65	3.37	6.95	4.92	0.65
		4:00	3.68	4.25	6.52	4.31	3.15	6.47	4.73	0.58
		5:00	3.74	3.36	5.88	5.73	3.72	5.23	4.61	0.46
		6:00	3.79	4.06	5.36	5.24	3.57	7.38	4.90	0.58
		7:00	3.83	3.60	6.80	4.79	3.19	7.10	4.88	0.69
		8:00	3.61	4.25	6.20	4.96	3.58	5.75	4.73	0.45
		9:00	4.56	5.19	6.55	5.9	1.8	7.32	5.22	0.79
	Blast 1	**10:00**	**9.85**	**9.76**	**11.91**	**10.04**	**7.67**	**9.26**	**9.75**	**0.56**
	Blast 2	**10:30**	**8.98**	**11.24**	**14.91**	**11.93**	**7.14**	**10.17**	**10.73**	**1.09**
	Blast 3	**11:00**	**9.13**	**11.13**	**12.67**	**11.81**	**7.17**	**10.15**	**10.34**	**0.81**
		**12:00**	**8.37**	**12.96**	**15.86**	**11.75**	**9.04**	**10.62**	**11.43**	**1.12**
		**13:00**	**10.73**	**4.31**	**14.22**	**11.81**	**9.71**	**12.38**	**10.53**	**1.39**
		**14:00**	**10.59**	**9.87**	**11.42**	**11.63**	**8.86**	**11.45**	**10.64**	**0.45**
		15:00	10.98	11.49	14.11	10.92	7.58	11.07	11.02	**0.85**
		16:00	11.66	12.11	13.98	10.10	9.27	10.86	11.33	**0.68**
		17:00	11.29	10.13	13.85	9.18	7.84	12.10	10.73	0.88
		18:00	11.96	11.68	15.02	10.56	7.61	11.66	11.41	0.98
		19:00	11.84	12.30	14.97	11.85	9.31	12.04	12.05	0.73
		20:00	9.92	11.19	14.39	10.65	7.62	11.48	10.88	0.90
		21:00	9.59	11.71	13.76	9.35	9.57	11.49	10.91	0.71
		22:00	11.59	11.10	14.98	10.86	9.55	11.71	11.63	0.74
		23:00	11.14	12.85	14.71	10.93	9.34	10.77	11.63	0.77

					ICP (mmHg)					
		Time			Animal number					
Day	Event	hh:mm	1	2	3	4	5	6	Average	SE
1		0:00	9.89	12.88	15.90	9.18	8.24	10.95	11.17	1.15
		1:00	9.99	12.11	14.26	10.03	8.03	11.39	10.97	0.87
		2:00	10.01	10.16	14.76	8.75	7.82	10.93	10.41	0.98
		3:00	11.45	10.33	14.78	9.19	8.22	12.27	11.04	0.96
		4:00	10.14	12.88	14.26	8.35	9.37	10.65	10.94	0.91
		5:00	11.86	10.64	14.82	8.19	8.58	12.41	11.08	1.02
		6:00	10.90	12.56	13.48	10.44	7.75	12.21	11.22	0.83
		7:00	12.49	11.95	13.35	9.73	9.29	12.47	11.55	0.67
		8:00	11.75	11.10	13.18	8.94	9.95	11.19	11.02	0.60
		9:00	12.20	11.57	15.63	8.67	8.53	12.71	11.55	1.09
		**10:00**	**11.22**	**10.19**	**15.65**	**8.23**	**7.73**	**13.48**	**11.08**	**1.25**
		**11:00**	**11.26**	**8.51**	**14.64**	**7.62**	**8.52**	**11.13**	**10.28**	**1.06**
		**12:00**	**12.98**	**11.25**	**14.83**	**8.04**	**8.52**	**12.34**	**11.33**	**1.08**
		**13:00**	**11.48**	**9.74**	**16.54**	**8.27**	**10.29**	**11.59**	**11.32**	**1.16**
		14:00	12.41	10.79	15.10	8.00	9.21	11.55	11.18	1.02
		15:00	12.70	10.81	12.13	8.85	8.58	11.53	10.77	0.70
		16:00	12.10	11.21	13.53	8.00	7.90	12.26	10.83	0.96
		17:00	11.54	9.97	13.62	8.10	9.53	11.44	10.70	0.78
		18:00	12.78	10.47	11.47	8.37	9.93	11.12	10.69	0.61
		19:00	12.35	11.23	11.89	8.38	9.74	11.89	10.91	0.63
		20:00	12.20	11.19	14.56	7.87	8.18	12.32	11.05	1.06
		21:00	12.55	11.12	13.72	8.71	8.54	12.47	11.19	0.88
		22:00	11.90	11.24	13.98	8.81	9.56	11.84	11.22	0.75
		23:00	11.32	10.13	12.12	8.11	9.02	10.90	10.27	0.61
2		0:00	11.60	11.21	13.41	8.08	8.37	12.56	10.87	0.89
		1:00	11.95	10.07	14.59	8.35	9.20	11.73	10.98	0.92
		2:00	12.56	11.26	12.15	8.36	7.90	11.59	10.64	0.82
		3:00	11.92	10.35	15.23	8.62	9.35	12.72	11.36	1.00
		4:00	11.52	11.17	12.85	8.71	9.53	12.13	10.99	0.64
		5:00	12.52	10.03	12.41	8.85	8.33	12.72	10.81	0.81
		6:00	12.15	10.51	12.89	8.47	8.07	12.77	10.81	0.88
		7:00	11.70	9.95	15.23	8.86	8.23	12.36	11.06	1.06
		8:00	12.66	10.00	13.78	7.95	8.97	11.88	10.87	0.92
		9:00	12.40	10.74	11.10	8.69	8.97	11.09	10.50	0.58
		**10:00**	**12.46**	**9.74**	**12.72**	**8.87**	**8.48**	**10.98**	**10.54**	**0.74**
		**11:00**	**12.47**	**9.59**	**11.73**	**8.02**	**7.95**	**11.88**	**10.27**	**0.83**
		**12:00**	**12.52**	**9.36**	**14.44**	**8.68**	**9.59**	**13.85**	**11.41**	**1.02**
		**13:00**	**12.38**	**10.48**	**9.89**	**8.92**	**7.85**	**13.63**	**10.53**	**0.88**
		14:00	12.54	10.15	12.71	8.37	8.63	13.33	10.95	0.89
		15:00	12.85	10.50	12.01	8.50	8.44	12.96	10.88	0.84
		16:00	12.31	10.56	12.38	8.22	8.53	13.98	11.00	0.94
		17:00	12.75	10.31	11.75	7.97	8.98	13.29	10.84	0.87
		18:00	12.76	10.84	12.76	8.87	8.38	13.89	11.25	0.92
		19:00	12.50	10.14	11.23	8.36	8.91	13.24	10.73	0.79

					ICP (mmHg)				
		Time			Animal number				
Day	Event	hh:mm	1	2	3	4	5	6	Average	SE
		20:00	12.44	10.24	12.32	8.16	9.31	12.94	10.90	0.80
		21:00	12.90	10.34	12.71	7.90	9.27	12.97	11.01	0.88
		22:00	12.55	10.02	12.26	8.90	9.87	12.89	11.08	0.69
		23:00	12.86	10.59	11.81	8.37	9.82	12.97	11.07	0.74
3		0:00	12.89	10.87	11.62	9.87	9.72	12.91	11.31	0.58
		1:00	12.32	10.26	11.38	8.77	9.71	12.90	10.89	0.65
		2:00	12.59	9.95	12.53	7.95	10.72	12.93	11.11	0.80
		3:00	12.47	10.85	11.46	10.86	10.89	12.95	11.58	0.37
		4:00	12.20	10.26	11.24	8.51	9.56	12.96	10.79	0.68
		5:00	11.94	10.43	12.23	8.17	10.91	12.98	11.11	0.70
		6:00	12.65	10.34	12.12	9.98	9.82	12.92	11.31	0.58
		7:00	12.40	10.54	12.27	8.15	9.95	12.97	11.05	0.75
		8:00	12.86	10.93	11.84	8.49	11.46	12.98	11.43	0.67
		9:00	12.96	10.80	12.80	8.44	11.59	12.96	11.59	0.72
		**10:00**	**10.78**	**9.43**	**15.94**	**10.31**	**11.32**	**12.61**	**11.73**	**0.95**
		**11:00**	**11.8**	**10.91**	**10.02**	**8.78**	**10.3**	**11.64**	**10.58**	**0.46**
		**12:00**	**12.54**	**10.29**	**12.4**	**10.71**	**9.84**	**12.5**	**11.38**	**0.50**
		**13:00**	**11.46**	**11.44**	**11.07**	**8.69**	**11.2**	**13.32**	**11.20**	**0.60**
		14:00	13.04	10.89	11.26	11.41	10.22	14.56	11.90	0.66
		15:00	12.14	11.25	11.31	10.95	11.09	14.69	11.90	0.58
		16:00	11.89	11.30	12.32	8.98	11.12	14.66	11.71	0.76
		17:00	13.00	11.09	11.90	11.03	10.89	13.76	11.95	0.49
		18:00	13.27	10.89	12.21	11.00	10.21	14.55	12.02	0.67
		19:00	12.06	11.25	11.19	9.34	10.65	14.76	11.54	0.74
		20:00	12.24	11.25	10.87	11.68	10.90	13.82	11.79	0.46
		21:00	13.03	11.07	12.39	11.94	10.49	13.40	12.05	0.46
		22:00	12.17	11.27	11.42	12.10	10.66	13.74	11.89	0.43
		23:00	12.92	11.28	10.96	11.66	10.43	14.56	11.97	0.62
4		0:00	12.15	11.17	13.31	11.55	11.29	13.73	12.20	0.44
		1:00	12.50	11.04	11.94	12.01	10.19	13.97	11.94	0.53
		2:00	12.00	10.89	11.93	11.73	10.78	13.55	11.81	0.41
		3:00	12.64	10.97	11.97	9.66	10.56	13.71	11.58	0.60
		4:00	12.39	11.33	12.02	11.96	10.24	14.16	12.02	0.53
		5:00	13.14	11.17	13.16	11.37	11.22	14.70	12.46	0.59
		6:00	13.07	11.06	12.62	10.47	11.32	13.41	11.99	0.49
		7:00	12.58	10.95	12.10	11.53	11.17	13.38	11.95	0.38
		8:00	12.92	11.27	11.31	10.96	10.35	13.46	11.71	0.49
		9:00	13.09	11.26	12.84	11.93	10.35	13.96	12.24	0.54
		**10:00**	**11.27**	**10.49**	**13**	**10.18**	**9.77**	**14.68**	**11.57**	**0.78**
		**11:00**	**13.33**	**10.11**	**12.51**	**9.2**	**10.48**	**15.19**	**11.80**	**0.93**
		**12:00**	**12.89**	**9.73**	**10.47**	**12.19**	**10.18**	**14.78**	**11.71**	**0.79**
		**13:00**	**11.85**	**9.55**	**12.15**	**10.49**	**8.85**	**13.02**	**10.99**	**0.66**
		14:00	11.46	10.46	11.76	11.49	8.94	14.43	11.42	0.74
		15:00	12.03	10.76	12.49	10.25	9.64	13.26	11.41	0.57

		Time	ICP (mmHg) Animal number							
Day	Event	hh:mm	1	2	3	4	5	6	Average	SE
		16:00	12.38	9.41	12.36	11.40	9.972	14.81	11.72	0.79
		17:00	12.81	8.44	12.23	11.42	9.893	13.26	11.34	0.76
		18:00	12.34	7.75	12.33	11.60	10.03	13.85	11.32	0.87
		19:00	11.48	8.03	11.64	12.02	10.38	13.28	11.14	0.73
		20:00	11.60	8.97	12.32	11.79	10.27	14.81	11.63	0.81
		21:00	11.22	8.89	12.40	12.27	10.13	13.66	11.43	0.70
		22:00	11.52	9.64	11.35	12.33	10.32	14.37	11.59	0.68
		23:00	12.92	7.21	11.80	12.69	10.36	13.81	11.47	0.98
5		0:00	13.29	7.89	11.53	11.72	9.64	14.60	11.45	0.99
		1:00	11.28	7.84	11.36	12.72	10.34	13.81	11.22	0.84
		2:00	12.48	8.83	11.26	13.26	9.97	13.80	11.60	0.79
		3:00	13.28	8.93	11.31	13.39	9.82	14.84	11.93	0.94
		4:00	13.06	9.37	11.24	13.52	9.81	13.99	11.83	0.81
		5:00	12.99	7.66	12.07	12.55	10.21	14.72	11.70	1.00
		6:00	11.56	8.19	11.39	13.75	9.80	13.91	11.43	0.91
		7:00	12.79	8.66	11.89	12.98	9.66	14.57	11.76	0.90
		8:00	11.37	7.25	11.99	14.19	10.10	14.15	11.51	1.07
		9:00	12.85	8.30	11.55	14.32	10.23	13.33	11.76	0.90
		**10:00**	**13.42**	**7.44**	**11.79**	**12.83**	**9.74**	**15.59**	**11.80**	**1.17**
		**11:00**	**13.26**	**7.42**	**12.6**	**14.55**	**9.39**	**15.96**	**12.20**	**1.31**
		**12:00**	**13.42**	**8.12**	**13.56**	**14.34**	**10.66**	**14.19**	**12.38**	**1.01**
		**13:00**	**12.56**	**6.83**	**10.41**	**13.85**	**10.43**	**14.47**	**11.43**	**1.15**
		14:00	11.86	8.56	11.23	13.04	9.98	13.98	11.44	0.81
		15:00	12.23	9.64	10.98	12.86	10.46	14.26	11.74	0.69
