# Supplementary material for: Effects of Exposure to Blast Overpressure on Intracranial Pressure and Blood-Brain Barrier Permeability in a Rat Model
Source: PLoS One. 2016 Dec 1;11(12):e0167510. doi: 10.1371/journal.pone.0167510 (PMC5132256; doi:10.1371/journal.pone.0167510)
Supplement: S3 File — (PDF) [file pone.0167510.s003.pdf]

Full 7 day telemetry ICP (intracranial pressure) data for 1x110 kPa group. The highlighted data is presented in Fig 1C.

			ICP (mmHg)							
		Time	Animal number							
Day	Event	hh:mm	1	2	3	4	5	6	Average	SE
-1	Baseline	9:00	1.23	5.26	4.35	4.26	5.43	6.32	4.48	0.72
		**10:00**	**4.37**	**3.54**	**5.58**	**4.25**	**4.72**	**4.34**	**4.47**	**0.27**
		**11:00**	**3.38**	**5.42**	**4.39**	**4.92**	**4.73**	**3.74**	**4.43**	**0.31**
		**12:00**	**2.99**	**5.9**	**6.56**	**4.88**	**5.36**	**5.26**	**5.16**	**0.49**
		**13:00**	**3.62**	**4.21**	**4.87**	**5.54**	**5.33**	**3.97**	**4.59**	**0.32**
		14:00	4.91	4.28	5.12	5.38	4.99	6.19	5.14	0.26
		15:00	5.00	4.01	5.10	5.14	4.83	4.64	4.79	0.17
		16:00	2.73	3.93	5.64	5.05	4.75	4.22	4.39	0.41
		17:00	3.38	4.86	5.56	4.84	5.30	4.75	4.78	0.31
		18:00	4.22	4.31	4.41	4.99	5.10	4.96	4.66	0.16
		19:00	2.50	4.98	5.90	4.40	5.03	6.22	4.84	0.54
		20:00	2.91	3.60	4.73	4.89	5.30	6.01	4.57	0.46
		21:00	3.87	4.09	5.50	5.37	5.30	3.94	4.68	0.32
		22:00	5.17	5.73	5.63	4.67	4.72	5.58	5.25	0.19
		23:00	2.23	3.94	6.38	4.99	4.81	4.25	4.43	0.56
0		0:00	4.62	5.70	6.29	4.79	5.34	4.30	5.17	0.30
		1:00	4.31	4.12	4.80	4.64	4.93	4.60	4.57	0.12
		2:00	4.11	5.37	4.85	4.51	4.79	4.55	4.70	0.17
		3:00	3.11	4.26	5.40	4.67	5.39	5.35	4.70	0.37
		4:00	4.90	4.33	5.27	4.52	5.18	4.40	4.77	0.17
		5:00	2.53	3.79	6.50	5.28	5.30	4.52	4.65	0.56
		6:00	2.07	5.17	5.74	5.21	4.97	5.97	4.86	0.58
		7:00	2.14	5.41	5.98	5.08	4.91	5.34	4.81	0.55
		8:00	2.02	5.29	5.57	5.42	5.32	5.09	4.79	0.56
		9:00	2.94	4.31	4.95	5.48	5.29	5.14	4.68	0.38
	Blast 1	**10:00**	**7.54**	**8.35**	**8.16**	**13.92**	**13.3**	**13.24**	**10.75**	**1.23**
		**11:00**	**7.6**	**6.76**	**9.12**	**15.39**	**15.23**	**14.03**	**11.36**	**1.62**
		**12:00**	**9.39**	**6.52**	**10.68**	**13.54**	**15.08**	**14.95**	**11.69**	**1.40**
		**13:00**	**7.69**	**7.31**	**8.68**	**16.58**	**11.69**	**15.09**	**11.17**	**1.61**
		**14:00**	**7.36**	**7.36**	**8.46**	**15.87**	**10.81**	**14.54**	**10.73**	**1.51**
		15:00	7.85	6.63	9.79	15.73	15.96	14.81	11.80	1.71
		16:00	7.91	6.98	10.18	15.80	14.56	14.79	11.70	1.56
		17:00	7.94	6.98	10.25	15.96	14.76	14.57	11.74	1.57
		18:00	7.95	6.99	10.30	16.33	15.43	14.59	11.93	1.65
		19:00	7.96	7.03	9.86	15.99	14.53	14.99	11.73	1.60
		20:00	7.99	7.51	11.45	16.91	14.34	14.91	12.19	1.58
		21:00	8.06	7.54	11.03	17.18	15.00	14.94	12.29	1.64
		22:00	8.07	7.61	12.12	17.15	15.34	14.59	12.48	1.61
		23:00	8.14	7.84	12.06	17.13	16.03	14.86	12.68	1.64

			ICP (mmHg)							
		Time			Animal number					
Day	Event	hh:mm	1	2	3	4	5	6	Average	SE
1		0:00	8.29	8.19	12.95	17.37	16.00	14.66	12.91	1.59
		1:00	8.31	8.25	13.45	17.53	15.00	14.71	12.88	1.55
		2:00	8.50	8.59	13.68	17.23	14.56	14.58	12.86	1.45
		3:00	8.53	8.63	14.84	18.65	15.23	14.82	13.45	1.65
		4:00	8.54	8.91	14.62	18.12	15.46	14.95	13.43	1.57
		5:00	8.79	9.16	15.32	17.86	16.03	14.79	13.66	1.54
		6:00	8.82	9.46	15.23	17.45	15.43	14.52	13.49	1.43
		7:00	8.88	9.46	16.18	18.44	15.49	14.85	13.88	1.57
		8:00	8.90	9.55	16.31	17.86	15.43	14.73	13.80	1.51
		9:00	8.91	9.65	13.72	18.85	15.76	14.59	13.58	1.54
		**10:00**	**7.49**	**9.23**	**15.13**	**18.76**	**14.46**	**14.46**	**13.26**	**1.69**
		**11:00**	**6.51**	**8.44**	**15.98**	**14.19**	**15**	**14.36**	**12.41**	**1.60**
		**12:00**	**8.24**	**9.42**	**18.03**	**17.24**	**15.54**	**14.74**	**13.87**	**1.67**
		**13:00**	**6.18**	**9.86**	**16.12**	**17.27**	**15.41**	**14.87**	**13.29**	**1.76**
		14:00	6.14	9.36	16.40	17.83	15.27	15.31	13.39	1.87
		15:00	6.16	9.69	16.55	17.75	15.29	15.50	13.49	1.85
		16:00	6.11	10.01	16.58	17.69	15.37	15.43	13.53	1.83
		17:00	6.17	10.26	16.66	16.53	15.72	15.94	13.55	1.77
		18:00	6.15	9.89	16.43	17.00	14.86	15.53	13.31	1.77
		19:00	6.13	10.61	16.83	16.96	15.82	15.48	13.64	1.78
		20:00	6.15	11.71	16.94	16.74	16.10	15.85	13.91	1.74
		21:00	6.14	11.76	17.53	17.23	16.17	15.68	14.08	1.80
		22:00	6.14	11.25	17.27	16.63	15.34	16.53	13.86	1.78
		23:00	6.13	12.08	17.87	16.51	16.56	14.98	14.02	1.77
2		0:00	6.12	12.54	17.00	15.46	16.59	14.79	13.75	1.66
		1:00	6.17	13.20	17.34	16.14	15.48	16.53	14.14	1.70
		2:00	6.16	13.11	18.37	17.83	16.75	16.68	14.82	1.89
		3:00	6.13	14.63	18.52	15.89	17.43	17.19	14.96	1.85
		4:00	6.17	14.23	17.98	15.38	16.85	17.13	14.62	1.78
		5:00	6.14	15.02	18.78	16.30	17.08	16.66	15.00	1.84
		6:00	6.14	15.20	18.46	15.19	16.28	16.27	14.59	1.76
		7:00	6.13	15.55	19.17	14.45	17.69	17.00	15.00	1.90
		8:00	6.16	14.00	18.46	14.31	16.86	16.54	14.39	1.78
		9:00	6.15	15.84	19.77	14.30	17.82	16.86	15.12	1.95
		**10:00**	**5.57**	**14.49**	**18.76**	**14.12**	**18.24**	**16.33**	**14.59**	**1.96**
		**11:00**	**6.41**	**14.95**	**19.31**	**10.59**	**17.09**	**16.78**	**14.19**	**1.96**
		**12:00**	**5.61**	**15.42**	**18.17**	**14.03**	**18.05**	**16.68**	**14.66**	**1.92**
		**13:00**	**5.35**	**17.06**	**20.17**	**11.5**	**17**	**17.26**	**14.72**	**2.20**
		14:00	5.46	16.16	20.17	13.30	17.53	17.40	15.00	2.11
		15:00	5.84	15.97	20.28	13.34	17.63	16.65	14.95	2.04
		16:00	5.86	15.91	20.32	14.31	17.33	17.01	15.12	2.02
		17:00	5.93	15.61	20.52	14.03	18.32	16.62	15.17	2.06
		18:00	5.55	15.56	20.40	13.32	17.16	16.98	14.83	2.08
		19:00	5.51	15.50	20.26	13.21	16.88	17.42	14.80	2.08

		Time			ICP (mmHg) Animal number					
Day	Event	hh:mm	1	2	3	4	5	6	Average	SE
		20:00	6.20	14.24	19.88	13.16	16.15	16.95	14.43	1.90
		21:00	5.50	14.21	19.79	14.20	16.01	16.65	14.39	1.97
		22:00	5.97	13.92	20.03	12.65	15.92	17.22	14.28	1.97
		23:00	6.17	13.74	20.42	14.31	15.88	16.73	14.54	1.93
3		0:00	5.40	13.64	19.95	13.54	16.02	16.93	14.25	2.02
		1:00	5.63	13.56	20.00	11.54	15.70	16.73	13.86	2.02
		2:00	6.04	13.43	19.88	13.12	14.91	17.41	14.13	1.93
		3:00	5.83	12.88	20.17	13.82	13.86	16.76	13.89	1.95
		4:00	5.62	12.50	19.49	11.46	14.71	17.13	13.48	1.98
		5:00	5.73	12.26	19.89	13.05	14.51	16.91	13.72	1.96
		6:00	5.90	12.07	20.40	10.13	13.89	17.09	13.25	2.09
		7:00	5.57	11.80	20.40	11.24	14.01	16.84	13.31	2.08
		8:00	6.09	11.52	19.58	12.34	14.00	17.01	13.42	1.91
		9:00	6.11	11.32	19.51	11.30	13.77	17.47	13.25	1.96
		**10:00**	**4.93**	**11.55**	**22.39**	**12.45**	**13.5**	**17.57**	**13.73**	**2.41**
		**11:00**	**5.71**	**11.33**	**20.75**	**11.66**	**12.87**	**16.76**	**13.18**	**2.10**
		**12:00**	**5.86**	**11.46**	**19.55**	**11.22**	**13.2**	**16.41**	**12.95**	**1.92**
		**13:00**	**6.26**	**10.27**	**20.79**	**13**	**14.97**	**16.48**	**13.63**	**2.06**
		14:00	5.54	11.39	20.53	11.28	11.78	16.35	12.81	2.08
		15:00	5.49	10.40	20.37	11.78	11.38	16.30	12.62	2.09
		16:00	6.00	10.90	20.33	11.44	11.39	16.26	12.72	2.02
		17:00	6.27	10.81	19.59	11.63	11.43	15.61	12.56	1.86
		18:00	5.69	10.57	17.88	11.55	11.19	15.29	12.03	1.71
		19:00	6.28	9.40	18.23	11.45	12.50	14.50	12.06	1.68
		20:00	5.52	10.27	19.75	12.39	12.00	14.71	12.44	1.93
		21:00	6.37	9.77	19.51	11.41	12.09	15.40	12.42	1.86
		22:00	5.55	9.73	19.08	10.87	10.78	14.61	11.77	1.88
		23:00	6.13	9.71	18.15	12.42	10.91	14.29	11.93	1.67
4		0:00	6.30	10.23	19.32	11.47	11.55	15.04	12.32	1.81
		1:00	6.34	9.23	19.46	11.56	12.47	13.72	12.13	1.81
		2:00	6.24	8.98	18.24	12.40	11.44	13.54	11.81	1.67
		3:00	5.99	8.86	18.12	10.75	12.04	12.34	11.35	1.66
		4:00	5.72	9.13	17.96	12.38	10.61	13.22	11.50	1.69
		5:00	6.28	8.19	17.81	12.49	11.26	13.17	11.53	1.65
		6:00	5.89	7.70	18.82	11.01	11.12	12.49	11.17	1.83
		7:00	6.39	6.46	18.67	10.95	11.92	13.00	11.23	1.87
		8:00	5.50	7.30	17.56	10.87	11.89	12.99	11.02	1.75
		9:00	5.90	7.14	17.64	10.91	10.76	12.77	10.85	1.71
		**10:00**	**5.69**	**7.77**	**17.48**	**9.2**	**9.59**	**12.81**	**10.42**	**1.70**
		**11:00**	**6.99**	**6.79**	**18.01**	**13.37**	**9.42**	**13.57**	**11.36**	**1.80**
		**12:00**	**5.21**	**6.18**	**18.97**	**15.77**	**10.65**	**13.4**	**11.70**	**2.21**
		**13:00**	**6.31**	**7.93**	**17.91**	**13.17**	**9.4**	**13.56**	**11.38**	**1.75**
		14:00	6.43	6.48	17.46	13.24	10.34	12.46	11.07	1.74
		15:00	6.34	6.56	17.49	13.49	9.86	13.46	11.20	1.80

			ICP (mmHg)							
		Time	Animal number							
Day	Event	hh:mm	1	2	3	4	5	6	Average	SE
		16:00	4.95	6.17	16.79	12.51	10.42	12.03	10.48	1.78
		17:00	6.18	5.93	16.78	12.51	10.24	11.34	10.50	1.67
		18:00	5.30	6.25	17.49	12.03	9.998	10.41	10.25	1.79
		19:00	6.53	5.89	15.27	11.56	9.814	11.54	10.10	1.43
		20:00	4.88	5.57	15.80	12.45	9.759	10.88	9.89	1.70
		21:00	4.67	6.36	12.89	10.81	10.46	9.69	9.15	1.25
		22:00	4.75	6.45	14.62	9.86	9.48	10.03	9.20	1.39
		23:00	6.23	5.99	12.87	10.29	9.14	8.95	8.91	1.05
5		0:00	4.59	5.79	14.64	10.15	9.40	11.23	9.30	1.50
		1:00	5.11	5.60	15.56	9.93	8.57	10.28	9.17	1.55
		2:00	5.30	6.14	14.50	9.90	8.48	10.83	9.19	1.37
		3:00	4.64	6.22	12.93	10.49	8.79	11.31	9.06	1.29
		4:00	5.90	5.75	13.51	9.46	8.00	8.68	8.55	1.16
		5:00	4.56	5.61	16.93	8.97	7.79	9.19	8.84	1.78
		6:00	4.97	5.88	15.49	9.19	7.78	9.34	8.77	1.52
		7:00	4.68	6.07	16.82	9.13	6.79	8.68	8.69	1.76
		8:00	4.63	5.87	14.53	8.84	7.73	8.69	8.38	1.40
		9:00	5.51	5.67	13.52	8.78	7.71	8.97	8.36	1.20
		**10:00**	**4.41**	**5.96**	**16.28**	**9.67**	**8.42**	**9.2**	**8.99**	**1.67**
		**11:00**	**5.16**	**6.23**	**12.8**	**8.85**	**6.18**	**10.39**	**8.27**	**1.20**
		**12:00**	**4.99**	**6.85**	**14.17**	**8.85**	**7.75**	**8.52**	**8.52**	**1.26**
		**13:00**	**5.04**	**5.69**	**15.05**	**7.53**	**9.43**	**8.59**	**8.56**	**1.47**
		14:00	4.96	6.46	14.43	8.49	8.91	9.16	8.74	1.32
		15:00	6.13	5.49	13.43	8.76	7.99	8.49	8.38	1.14
