# Supplementary material for: Effects of Exposure to Blast Overpressure on Intracranial Pressure and Blood-Brain Barrier Permeability in a Rat Model
Source: PLoS One. 2016 Dec 1;11(12):e0167510. doi: 10.1371/journal.pone.0167510 (PMC5132256; doi:10.1371/journal.pone.0167510)
Supplement: S4 File — (PDF) [file pone.0167510.s004.pdf]

Full 7 day telemetry ICP (intracranial pressure) data for 3x110 kPa group. The highlighted data is presented in Fig 1D.

			ICP (mmHg)							
		Time	Animal number							
Day	Event	hh:mm	1	2	3	4	5	6	Average	SE
-1	Baseline	9:00	1.23	5.26	4.35	4.26	5.43	6.32	4.48	0.72
		**10:00**	**2.96**	**4.21**	**8.43**	**6.03**	**6.02**	**6.96**	**5.77**	**0.79**
		**11:00**	**2.36**	**6.08**	**9.22**	**6.14**	**6.02**	**6.40**	**6.04**	**0.89**
		**12:00**	**1.76**	**5.20**	**8.23**	**5.94**	**6.02**	**7.23**	**5.73**	**0.91**
		**13:00**	**1.72**	**7.03**	**9.23**	**5.35**	**6.47**	**7.05**	**6.14**	**1.02**
		14:00	2.17	5.16	5.46	5.91	5.29	8.31	5.38	0.80
		15:00	2.49	6.46	6.43	5.63	6.42	7.16	5.77	0.68
		16:00	3.28	4.23	4.50	5.87	5.17	8.94	5.33	0.81
		17:00	3.19	5.48	5.43	5.80	6.77	8.43	5.85	0.70
		18:00	3.54	7.21	6.86	5.82	5.41	7.28	6.02	0.58
		19:00	3.94	6.02	4.84	6.07	6.84	8.55	6.04	0.65
		20:00	2.77	5.97	5.48	4.55	5.31	7.67	5.29	0.66
		21:00	3.86	4.95	5.77	4.49	6.74	6.81	5.44	0.49
		22:00	4.32	4.85	4.99	5.21	5.00	8.43	5.47	0.60
		23:00	3.55	7.29	6.59	5.85	5.18	7.42	5.98	0.60
0		0:00	2.31	5.54	6.49	4.60	5.99	9.15	5.68	0.92
		1:00	2.58	7.04	4.11	4.86	5.23	8.33	5.36	0.84
		2:00	2.43	5.14	4.53	5.37	4.95	8.81	5.21	0.84
		3:00	3.86	5.19	4.15	5.97	6.76	7.09	5.50	0.55
		4:00	3.39	5.01	5.29	4.38	6.77	9.21	5.68	0.84
		5:00	2.35	4.55	4.91	4.46	6.20	7.93	5.07	0.76
		6:00	3.47	6.67	5.57	5.37	6.58	6.45	5.69	0.50
		7:00	2.63	6.71	6.35	4.54	5.52	7.11	5.48	0.68
		8:00	3.70	6.74	6.41	5.85	5.45	6.50	5.77	0.46
		9:00	4.35	5.96	5.48	7.13	6.02	9.86	6.47	0.77
	Blast 1	**10:00**	**14.18**	**8.38**	**19.80**	**18.55**	**12.29**	**11.57**	**14.13**	**1.78**
	Blast 2	**10:30**	**10.84**	**14.68**	**19.82**	**19.02**	**12.69**	**12.09**	**14.86**	**1.53**
	Blast 3	**11:00**	**11.32**	**10.19**	**18.41**	**21.13**	**13.31**	**16.32**	**15.11**	**1.74**
		**12:00**	**13.61**	**11.58**	**21.27**	**24.22**	**11.81**	**15.65**	**16.36**	**2.14**
		**13:00**	**14.38**	**11.46**	**20.68**	**19.45**	**12.03**	**17.67**	**15.95**	**1.59**
		**14:00**	**13.46**	**10.98**	**21.33**	**18.47**	**11.83**	**20.49**	**16.09**	**1.86**
		15:00	13.24	12.34	20.49	17.79	12.79	20.46	16.19	1.57
		16:00	12.49	10.98	19.98	17.46	11.64	18.43	15.16	1.59
		17:00	12.86	13.28	20.10	19.08	15.27	19.05	16.61	1.31
		18:00	15.74	13.64	23.98	23.19	12.97	21.21	18.46	2.01
		19:00	14.48	14.59	23.03	17.80	12.70	21.66	17.38	1.72
		20:00	12.36	15.88	21.39	20.59	16.07	20.01	17.72	1.44
		21:00	15.57	16.38	23.97	21.16	14.85	20.50	18.74	1.49
		22:00	12.17	16.58	19.25	23.69	15.20	19.75	17.77	1.64
		23:00	13.20	17.09	19.00	19.08	14.73	20.19	17.22	1.12

			ICP (mmHg)							
		Time	Animal number							
Day	Event	hh:mm	1	2	3	4	5	6	Average	SE
1		0:00	12.13	16.39	19.90	19.98	11.36	21.97	16.95	1.81
		1:00	13.71	16.30	22.15	26.04	13.38	20.08	18.61	2.05
		2:00	13.53	13.14	22.16	18.35	15.90	20.05	17.19	1.48
		3:00	15.14	13.84	20.71	18.78	17.81	21.13	17.90	1.20
		4:00	13.45	14.66	21.83	27.48	12.82	18.51	18.13	2.33
		5:00	12.91	14.66	22.08	19.93	17.05	18.10	17.45	1.37
		6:00	15.19	20.72	20.64	17.05	13.51	21.46	18.10	1.36
		7:00	12.72	21.27	20.84	24.27	11.56	19.90	18.43	2.08
		8:00	15.23	20.20	23.29	26.54	13.27	19.68	19.70	2.01
		9:00	15.33	19.86	19.34	24.88	17.10	18.49	19.17	1.32
		**10:00**	**15.61**	**20.59**	**23.36**	**28.52**	**17.51**	**19.39**	**20.83**	**1.88**
		**11:00**	**16.03**	**20.85**	**23.06**	**28.23**	**16.81**	**16.89**	**20.31**	**1.94**
		**12:00**	**15.48**	**19.98**	**23.75**	**27.78**	**16.66**	**26.75**	**21.73**	**2.11**
		**13:00**	**15.65**	**21.15**	**24.94**	**28.86**	**19.03**	**15.82**	**20.91**	**2.13**
		14:00	14.69	20.77	23.83	24.70	19.00	22.66	20.94	1.51
		15:00	14.64	19.55	23.16	26.28	17.53	23.95	20.85	1.79
		16:00	14.36	20.63	21.72	26.23	17.59	23.02	20.59	1.70
		17:00	14.41	19.30	21.75	26.44	18.75	23.26	20.65	1.69
		18:00	13.94	19.79	22.06	24.48	17.00	23.17	20.07	1.63
		19:00	13.68	19.73	22.20	27.18	18.14	20.81	20.29	1.83
		20:00	13.31	19.17	20.65	26.66	18.01	21.54	19.89	1.79
		21:00	13.59	20.95	23.48	26.59	18.82	21.81	20.87	1.80
		22:00	13.87	19.71	23.21	25.91	17.56	21.71	20.33	1.74
		23:00	13.42	19.48	22.77	26.78	18.20	21.64	20.38	1.85
2		0:00	15.54	19.92	19.42	24.16	18.50	22.98	20.09	1.27
		1:00	15.68	19.76	19.48	22.89	17.62	21.78	19.53	1.08
		2:00	14.62	19.12	18.29	22.34	18.46	18.17	18.50	1.01
		3:00	14.31	19.86	19.09	22.46	18.69	19.47	18.98	1.08
		4:00	14.94	20.50	19.42	20.46	18.25	18.43	18.67	0.84
		5:00	14.61	19.56	18.84	18.85	17.55	16.12	17.59	0.78
		6:00	13.26	20.27	22.24	17.63	18.70	17.07	18.20	1.25
		7:00	13.39	19.45	18.97	18.18	18.85	16.83	17.61	0.92
		8:00	13.18	19.32	17.35	19.31	18.36	15.11	17.10	1.01
		9:00	12.48	20.60	19.77	18.59	17.60	15.64	17.45	1.22
		**10:00**	**13.98**	**19.51**	**18.80**	**19.06**	**17.37**	**14.67**	**17.23**	**0.97**
		**11:00**	**14.55**	**18.76**	**18.85**	**17.74**	**18.38**	**14.52**	**17.13**	**0.84**
		**12:00**	**13.62**	**19.45**	**18.76**	**21.66**	**17.75**	**14.10**	**17.56**	**1.28**
		**13:00**	**15.63**	**19.08**	**18.35**	**24.02**	**18.00**	**14.70**	**18.30**	**1.34**
		14:00	15.32	18.96	19.07	22.52	18.37	13.36	17.93	1.31
		15:00	11.90	18.82	18.15	22.41	18.60	12.16	17.01	1.69
		16:00	15.60	18.44	17.45	22.23	18.67	12.20	17.43	1.37
		17:00	15.53	18.34	18.47	22.42	18.28	11.03	17.34	1.55
		18:00	10.94	17.60	18.65	23.00	18.45	11.36	16.67	1.91
		19:00	11.15	17.36	17.94	22.27	18.28	13.22	16.70	1.62

			ICP (mmHg)							
		Time	Animal number							
Day	Event	hh:mm	1	2	3	4	5	6	Average	SE
		20:00	13.21	16.97	17.97	22.97	18.16	13.85	17.19	1.44
		21:00	12.91	15.82	17.94	22.16	18.86	10.30	16.33	1.74
		22:00	13.96	15.77	17.42	21.03	18.86	12.60	16.61	1.28
		23:00	13.43	16.61	18.80	21.69	18.12	10.84	16.58	1.59
3		0:00	11.81	15.12	17.13	21.93	18.70	12.35	16.17	1.58
		1:00	13.46	15.05	17.57	21.69	18.18	12.97	16.49	1.35
		2:00	13.58	14.97	16.29	21.46	18.42	12.14	16.14	1.38
		3:00	12.84	15.13	17.56	21.18	18.22	11.10	16.00	1.52
		4:00	12.67	13.84	16.43	20.90	18.41	10.11	15.39	1.62
		5:00	13.32	13.37	16.32	19.65	18.57	11.83	15.51	1.29
		6:00	13.57	13.89	16.00	19.88	18.63	11.29	15.54	1.33
		7:00	11.20	12.77	16.10	21.59	18.37	10.27	15.05	1.81
		8:00	11.08	13.99	16.28	19.76	18.53	11.89	15.26	1.44
		9:00	11.01	12.06	16.25	19.62	18.17	12.79	14.98	1.44
		**10:00**	**10.85**	**11.77**	**16.77**	**20.33**	**20.60**	**11.46**	**15.30**	**1.85**
		**11:00**	**11.17**	**11.14**	**16.14**	**20.10**	**18.47**	**9.42**	**14.41**	**1.81**
		**12:00**	**10.40**	**8.70**	**13.70**	**17.52**	**18.11**	**12.39**	**13.47**	**1.54**
		**13:00**	**10.58**	**12.02**	**17.02**	**17.31**	**17.89**	**14.13**	**14.83**	**1.25**
		14:00	10.44	9.92	16.05	17.31	18.04	11.30	13.85	1.51
		15:00	9.73	11.34	16.05	17.54	17.66	11.00	13.89	1.47
		16:00	9.57	11.21	15.88	18.55	17.19	10.96	13.89	1.54
		17:00	9.28	10.31	15.72	17.13	17.92	11.58	13.66	1.52
		18:00	9.10	7.56	15.26	17.34	18.07	10.99	13.06	1.81
		19:00	8.62	10.00	13.49	18.51	18.14	11.32	13.35	1.71
		20:00	8.56	9.83	13.20	16.89	17.47	11.44	12.90	1.50
		21:00	8.53	10.23	14.32	16.10	17.62	11.53	13.06	1.44
		22:00	7.74	9.72	14.09	17.14	16.68	11.04	12.73	1.57
		23:00	7.69	9.34	13.13	16.59	17.57	11.62	12.66	1.60
4		0:00	7.67	10.20	12.58	17.14	15.14	10.94	12.28	1.41
		1:00	7.62	8.78	12.01	16.41	16.68	11.53	12.17	1.54
		2:00	8.28	7.17	11.67	16.26	15.88	11.27	11.76	1.53
		3:00	7.40	7.79	11.56	16.64	15.84	11.29	11.75	1.59
		4:00	7.29	8.27	11.49	16.86	15.09	11.40	11.73	1.52
		5:00	6.21	8.44	11.99	15.86	15.77	11.27	11.59	1.58
		6:00	7.59	7.60	11.81	14.18	16.69	11.15	11.50	1.47
		7:00	6.56	7.76	11.80	15.82	16.20	11.34	11.58	1.62
		8:00	6.43	8.69	10.98	14.13	14.95	11.15	11.06	1.31
		9:00	5.90	7.39	11.12	14.28	15.46	10.86	10.84	1.52
		**10:00**	**6.24**	**8.63**	**13.71**	**16.58**	**16.24**	**11.52**	**12.15**	**1.70**
		**11:00**	**4.69**	**8.48**	**11.82**	**16.68**	**15.90**	**10.95**	**11.42**	**1.84**
		**12:00**	**4.54**	**7.99**	**11.61**	**16.33**	**16.08**	**13.38**	**11.66**	**1.90**
		**13:00**	**3.52**	**5.19**	**11.05**	**12.19**	**15.16**	**12.95**	**10.01**	**1.88**
		14:00	5.44	7.92	11.20	15.53	15.38	11.15	11.10	1.63
		15:00	5.03	7.95	10.62	14.26	15.01	11.12	10.67	1.54

		Time	ICP (mmHg) Animal number							
Day	Event	hh:mm	1	2	3	4	5	6	Average	SE
		16:00	5.36	7.03	9.91	15.59	15.21	11.13	10.71	1.70
		17:00	5.32	6.90	10.14	15.07	15.59	11.05	10.68	1.70
		18:00	5.16	8.59	11.11	13.07	15.54	10.29	10.63	1.47
		19:00	5.22	7.72	11.30	13.04	14.33	9.87	10.25	1.38
		20:00	5.03	5.68	11.19	13.91	15.36	10.71	10.31	1.72
		21:00	5.55	6.62	10.78	14.98	15.32	10.90	10.69	1.66
		22:00	5.17	6.86	10.46	12.97	15.44	10.27	10.20	1.55
		23:00	5.39	8.29	10.84	15.30	14.57	11.42	10.97	1.53
5		0:00	5.05	5.05	11.00	14.54	14.82	11.24	10.28	1.78
		1:00	5.08	8.54	10.77	12.53	13.17	11.09	10.20	1.22
		2:00	5.21	7.14	11.06	11.40	14.36	10.76	9.99	1.34
		3:00	5.58	6.85	10.16	10.20	12.21	11.41	9.40	1.07
		4:00	5.08	7.88	11.15	10.41	12.14	10.90	9.59	1.07
		5:00	4.85	7.95	10.16	13.23	12.68	11.13	10.00	1.29
		6:00	5.62	5.93	11.15	13.40	12.46	10.16	9.79	1.35
		7:00	4.97	6.73	10.77	10.33	12.39	10.44	9.27	1.15
		8:00	5.20	7.14	10.50	10.91	11.56	10.91	9.37	1.05
		9:00	5.59	5.73	10.08	10.45	11.49	11.49	9.14	1.12
		**10:00**	**3.99**	**6.32**	**11.22**	**10.27**	**12.67**	**12.80**	**9.54**	**1.47**
		**11:00**	**5.41**	**6.24**	**11.36**	**12.28**	**13.72**	**7.21**	**9.37**	**1.43**
		**12:00**	**2.24**	**6.00**	**12.17**	**10.79**	**11.96**	**11.87**	**9.17**	**1.68**
		**13:00**	**1.61**	**4.87**	**11.64**	**12.22**	**12.56**	**13.63**	**9.42**	**2.02**
		14:00	3.16	5.46	11.65	11.59	11.49	12.34	9.28	1.60
		15:00	2.98	6.29	11.03	11.46	12.76	12.21	9.46	1.60
